# Publisher Correction: Mining multi-center heterogeneous medical data with distributed synthetic learning

**DOI:** 10.1038/s41467-023-41835-0

**Published:** 2023-10-24

**Authors:** Qi Chang, Zhennan Yan, Mu Zhou, Hui Qu, Xiaoxiao He, Han Zhang, Lohendran Baskaran, Subhi Al’Aref, Hongsheng Li, Shaoting Zhang, Dimitris N. Metaxas

**Affiliations:** 1 Department of Computer Science, Rutgers University, Piscataway, NJ USA; 2 SenseBrain Research, Princeton, NJ USA; 3 Shanghai Artificial Intelligence Laboratory, Shanghai, China; 4 Department of Cardiovascular Medicine, National Heart Centre Singapore, and Duke-National University Of Singapore, Singapore, Singapore; 5 Department of Medicine, Division of Cardiology, University of Arkansas for Medical Sciences, Little Rock, AR USA; 6 Chinese University of Hong Kong, Hong Kong SAR, China; 7 Centre for Perceptual and Interactive Intelligence (CPII), Hong Kong SAR, China; 8 SenseTime, Shanghai, China

**Keywords:** Machine learning, Data acquisition, Data mining, Medical imaging

Correction to: *Nature Communications* 10.1038/s41467-023-40687-y, published online 07 September 2023

In this article, Mu Zhou should have been denoted as an equally contributing author. The original article has been corrected.

