# Supplementary material for: Mining multi-center heterogeneous medical data with distributed synthetic learning
Source: Nat Commun. 2023 Sep 7;14:5510. doi: 10.1038/s41467-023-40687-y (PMC10484909; doi:10.1038/s41467-023-40687-y)
Supplement: Supplementary file 1 — Supplementary Information [file 41467_2023_40687_MOESM1_ESM.pdf]

Mining Multi-Center Heterogeneous Medical Data with Distributed Synthetic Learning: Supplementary Material

Qi Chang[1,9], Zhennan Yan[2,9], Mu Zhou[2,3,9], Hui Qu[1], Xiaoxiao He[1], Han Zhang[1], Lohendran Baskaran[4], Subhi Al'Aref[5], Hongsheng Li[6,7,10], Shaoting Zhang[3,7,8,10] and Dimitris N. Metaxas[1,10]

[1] *Department of Computer Science, Rutgers University, Piscataway, New Jersey, USA*
[2] *SenseBrain Research, Princeton, NJ, USA*
[3] *Shanghai Artificial Intelligence Laboratory, Shanghai, China*
[4] *Department of Cardiovascular Medicine, National Heart Centre Singapore, and Duke-National University Of Singapore*
[5] *Department of Medicine, Division of Cardiology. University of Arkansas for Medical Sciences, Little Rock, AR, USA*
[6] *Chinese University of Hong Kong, Hong Kong SAR, China*
[7] *Centre for Perceptual and Interactive Intelligence (CPII), Hong Kong SAR, China*
[8] *SenseTime, Shanghai, China*
[9] *These authors contributed equally: Qi Chang, Zhennan Yan and Mu Zhou*
[10] *Corresponding authors: Dimitris N. Metaxas (dnm@cs.rutgers.edu), Shaoting Zhang (zhangshaoting@pjlab.org.cn), and Hongsheng Li (hsli@ee.cuhk.edu.hk).*

Here, we provide more experimental results, visualizations, and discussions for our study.

1 Supplementary results on each experiment with the box plots

Supplementary Figure 1 shows the box plots of the DICE, HD95, SD, and ACC scores across four experiments. These results have been summarized in the tables in the Results section of the manuscript. DSL demonstrates superior performance compared to other models trained with local data or other distributed GAN methods.

2 Supplementary results for FID and Dist-FID metrics

The following three charts in Supplementary Figure 2 depict the best epoch model selection strategy. The Dist-FID shows the consistent best epoch score compared to the original FID. By contrast, the rest selection strategy could not accurately reflect the optimized model. FID_1% and FID_5% scores show the FID using the given percentage subset of all data. The FID of each source shows the scores calculated from the given local dataset only. The black arrows indicate the optimized epoch for FID and Dist-FID. The FID is the ideal score to identify the optimized epoch generative model with the best synthetic image quality. However, simply applying the FID inception feature calculation to the distributed private datasets tends to be impractical since the data privacy restriction and significant bandwidth of inception feature accumulation for all real-synthetic image pairs. It's also worth noting that the more heterogeneity there is in the cohort, the greater fluctuation between different medical image centers. No matter the heterogeneity of each experiment, Dist-FID is always consistent with the FID score which is calculated from all data together.

3 Supplementary results of the variation of synthetic images by DSL

We show examples by using such test-time augmentation (TTA) in Supplementary Figures 3, 4, and 5. DSL's implementation provides random noise in the form of dropout by applying several layers of the generator at both training and testing. Therefore, there is a minor stochasticity in the synthetic images when repeating the same input. However, we can make DSL to generate more diverse images by applying random transformations to the input in testing, such as random scaling, shift, flip, and so on. We can see that by applying random TTA on the input label images, the generator produces varied contexts in many detailed areas while keeping a similar image quality. Due to the random transformations of the input, the synthetic images may have slightly different scales and positions (flip if any was reversed for better visualization).

4 Examples of the heterogeneous images over different data centers

We collected three categories of datasets to evaluate our method, including 1) multi-center cardiac computed tomography angiography (CTA); 2) multi-modality brain magnetic resonance imaging (MRI); 3) multi-organ histopathology images. In terms of data heterogeneous, Supplementary Figures 6, 7, and 8 show the differences in the appearance of some real data samples among

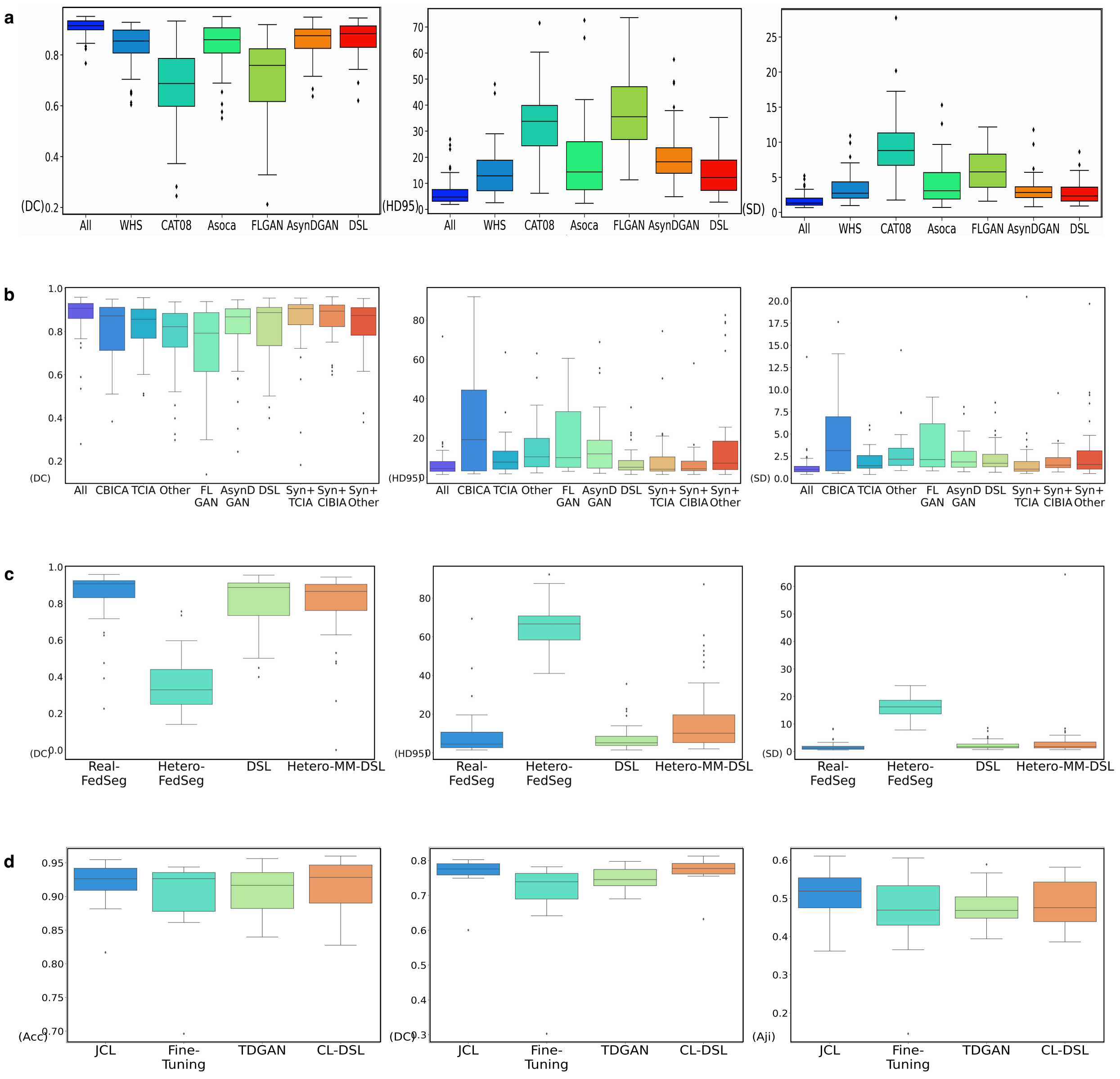

Supplementary Figure 1: The box plots of results in different experiments. **a** The segmentation results on the cardiac CTA images (N=54). **b** The segmentation results on the multi-modality BraTS dataset (N=42). **c** The segmentation results on the missing modality brain images (N=42). **d** The results of continual learning on pathology images (N=8). DC: Dice score, HD95: 95% Hausdorff distance, SD: average surface distance, ACC: accuracy, AJI: aggregated Jaccard index. All box plots include the median line, the box denotes the interquartile range (IQR), whiskers denote the rest of the data distribution and outliers are denoted by points greater than $\pm 1.5 \times$IQR.

multiple centers and the intensity histograms of all data samples in each data center.

5 Algorithm of learning DSL

The learning process of DSL is summarized in Supplementary Algorithm 1. All the symbols and equations are defined in the Methods section of the manuscript.

Supplementary Algorithm 1 Algorithm of learning DSL

Require: the number of discriminators (clients) N, initial generator and discriminator parameters θ_G, θ_{D_j} ($j \in \{1, ..., N\}$), minibatch size m, number of iterations R_{max}, sample distribution $s_j(x)$ and size of dataset $|S_j|$ at node j

for $j = 1 : N$ **do**
  D_j sends its available auxiliary variables $\{x_i^j | i = 1, ..., |S_j|\}$ to generator G
end for
for $r = 1 : R_{max}$ **do**
  for $j = 1 : N$ **do**
    G samples minibatch of m variables $x_i^j \sim s_j(x)$ as input to generate m fake data $\hat{y}_i^j = G(x_i^j)$, and sends them to node D_j
    D_j finds corresponding samples y_i^j and updates θ_{D_j} according to the loss L_{D_j} (Eq. 2)
    fix θ_{D_j} to compute gradients $\nabla_{\hat{y}^j}$ based on L_{G_j} (Eq. 4) and send to G
  end for
  for $j = 1 : N$ **do**
    G receives gradients $\nabla_{\hat{y}^j}$ from D_j
  end for
  G aggregates gradients (Eq. 5) with $\pi_j = |S_j|$ and updates θ_G
end for
return The learned models θ_G and θ_{D_j} ($j \in \{1, ..., N\}$)

6 Supplementary results for MM-DSL classification as downstream task

In Supplementary Table 1, we present the classification results in order to validate the generalization ability of DSL. We verify the MM-DSL synthetic image quality with a downstream binary classification task of image-based tumor region recognition. The accuracy of MM-DSL is 0.897, which is significantly higher than the other synthetic-image-based methods. For instance, MM-DSL outperforms FLGAN by 27%. MM-DSL also achieves better performance than the segmentation model trained by using a small-scale real dataset (Real-OTHER), which only obtains an accuracy of 0.74%.

Supplementary Table 1: Quantitative results of the binary classification task. All methods learn from multi-modality brain MRI for tumor image recognition. In the first column, 'Real-' indicates the model trained from original real images, otherwise, the model is trained from synthetic images. Real-All merges together all data of CBICA, TCIA, and OTHER. The classification model trained from MM-DSL's synthetic data outperforms the other generative methods and is comparable to the models trained from real data.

Data/Method	Accuracy ↑	Sensitivity↑	Specificity↑
Real-All	0.923	0.895	0.944
Real-CBICA	0.874	0.798	0.929
Real-TCIA	0.89	0.855	0.916
Real-OTHER	0.743	0.681	0.787
FLGAN	0.653	0.932	0.460
FedMed-GAN	0.745	0.885	0.644
AsynDGAN	0.84	0.783	0.881
MM-DSL	0.897	0.853	0.928

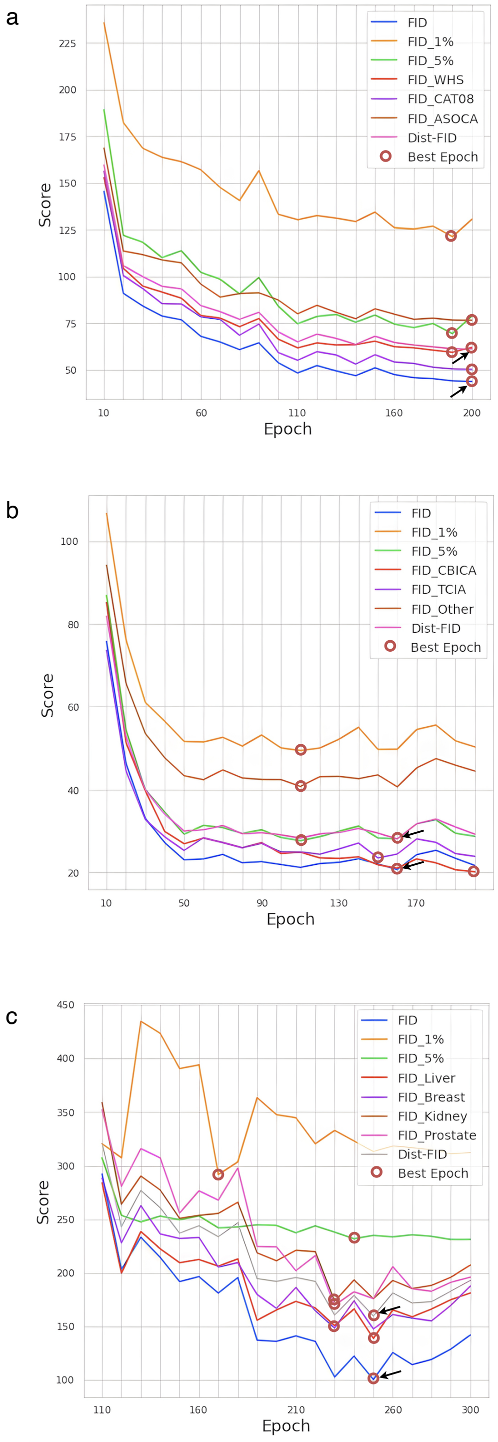

Supplementary Figure 2: The complete set of FID and Dist-FID scores in three experiments. **a** Results for the DSL with the cardiac dataset. **b** Results for the multi-modality DSL with the Brain BraTS dataset. **c** Results for the continual-learning DSL with the Nuclei dataset. The red circles indicate the best epoch found by each measurement, while the arrows show the consistency in the best epoch of FID and Dist-FID scores.

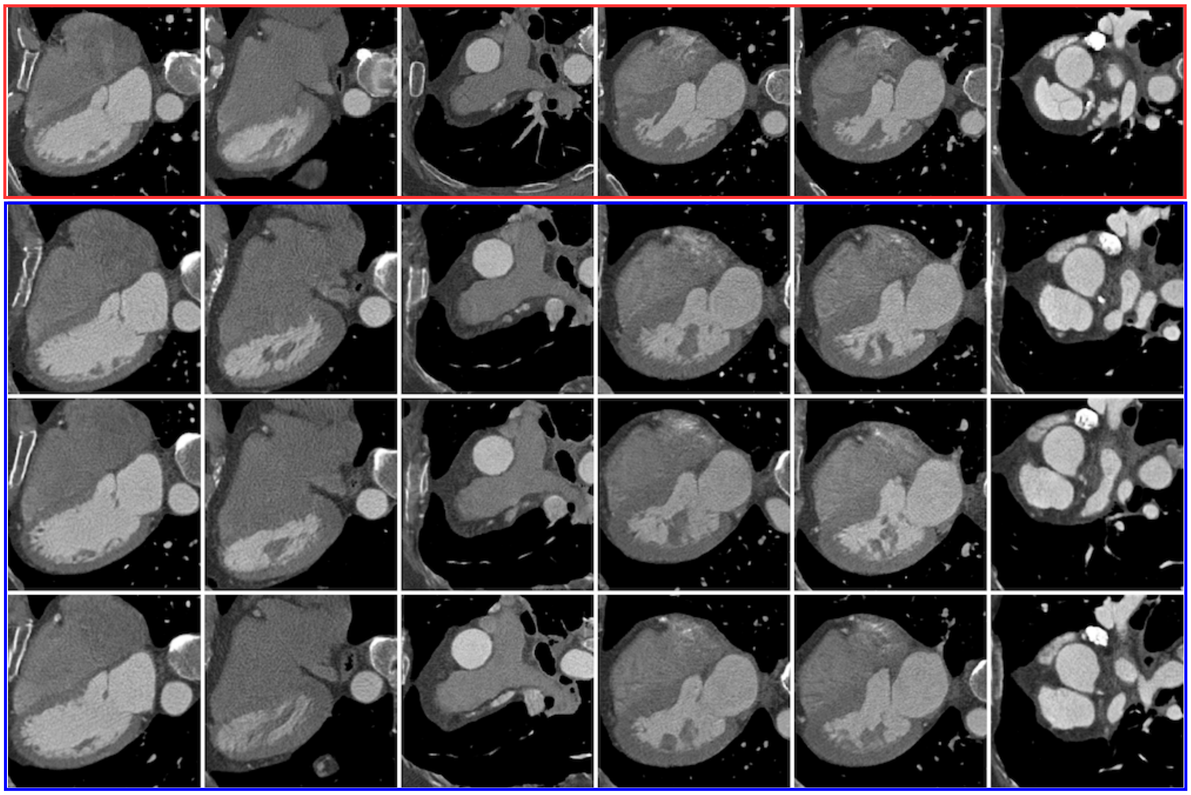

Supplementary Figure 3: Additional variation of synthetic cardiac CTA images. The first row shows six real cardiac CT images, and the following three rows show the corresponding synthetic images generated by DSL by repeating independently three times with different random transforms (scaling and shift) on the inputs.

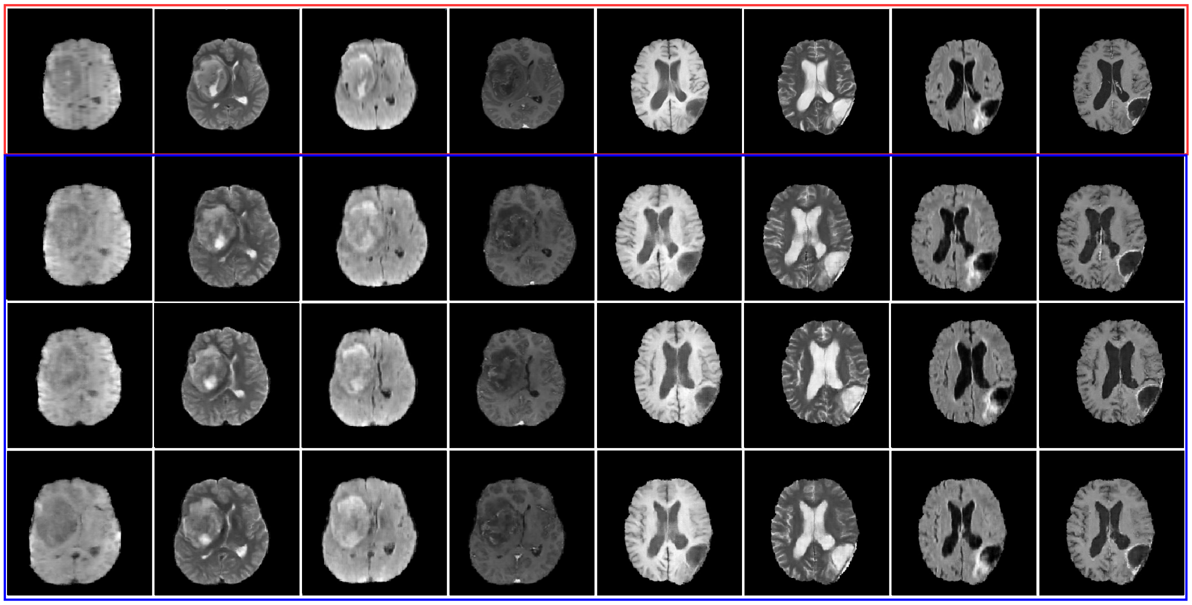

Supplementary Figure 4: Additional variation of synthetic multi-modality brain MR images. The first row shows the real brain MR images (including four modalities T1, T2, Flair, T1c) of two cases, and the following three rows show the corresponding synthetic images generated by DSL by repeating independently three times with different random transforms (scaling, shift, and flip) on the inputs.

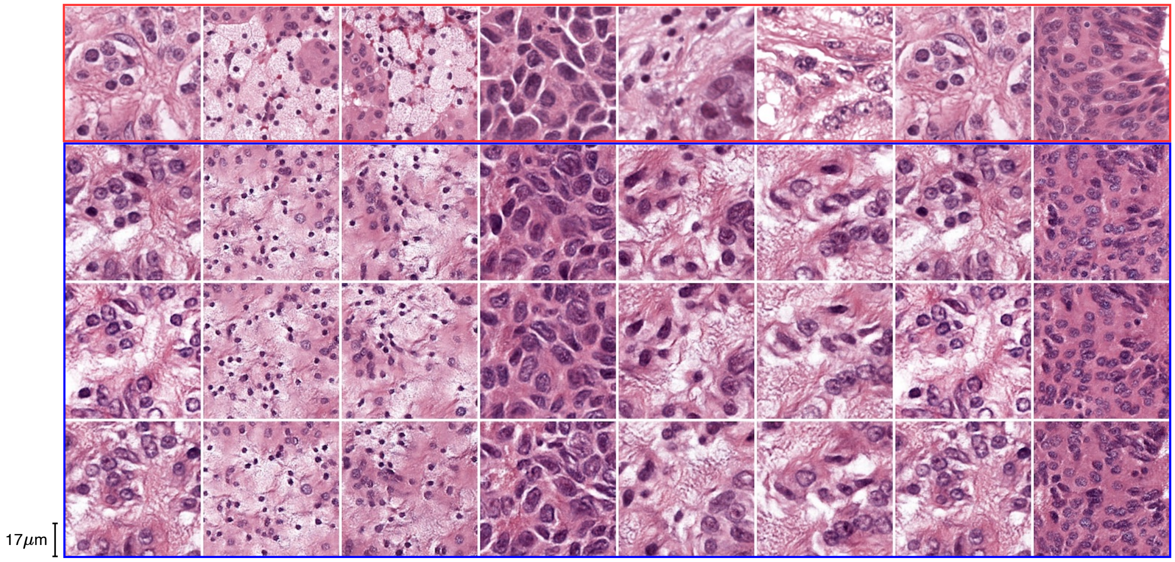

Supplementary Figure 5: Additional variation of synthetic histopathology images. The first row shows eight real histopathology image patches, and the following three rows show the corresponding synthetic images generated by DSL by repeating independently three times with different random transforms (scaling, shift, and flip) on the inputs.

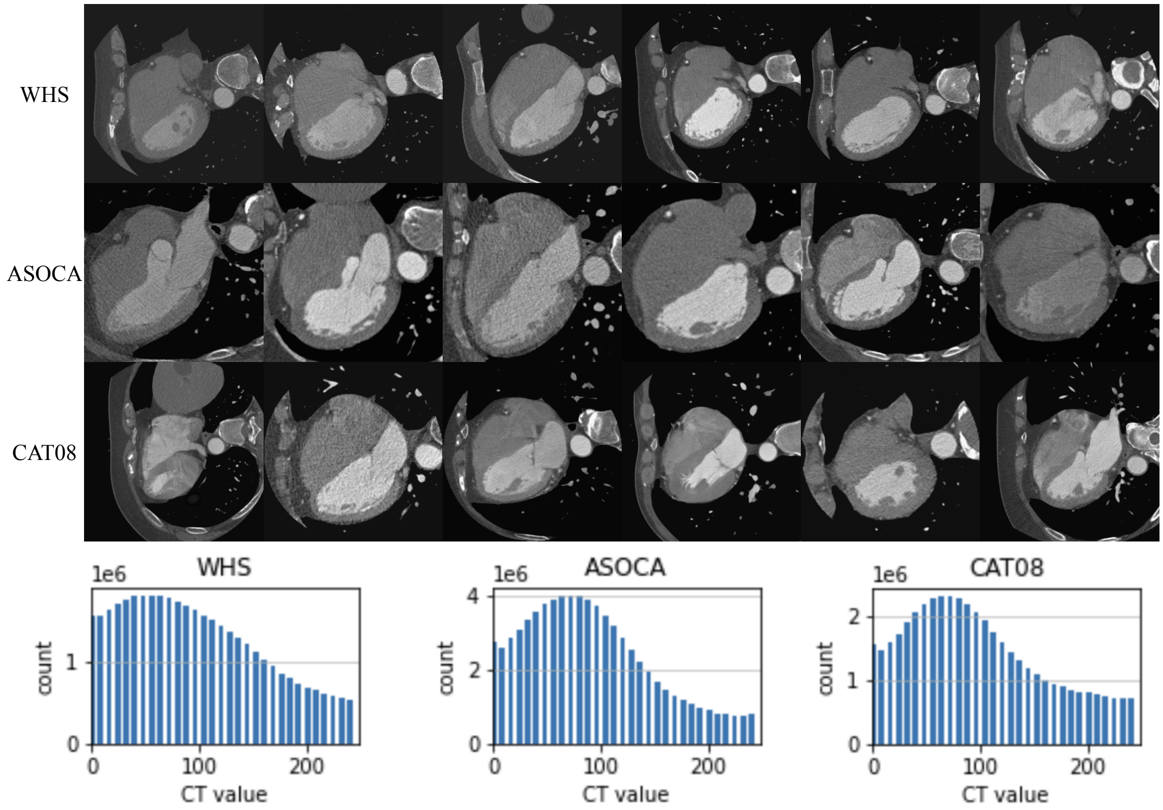

Supplementary Figure 6: Data heterogeneous of cardiac CTA datasets. Randomly selected real image samples (6 samples per center) and intensity histogram charts from three different CTA data centers that are used in the experiments.

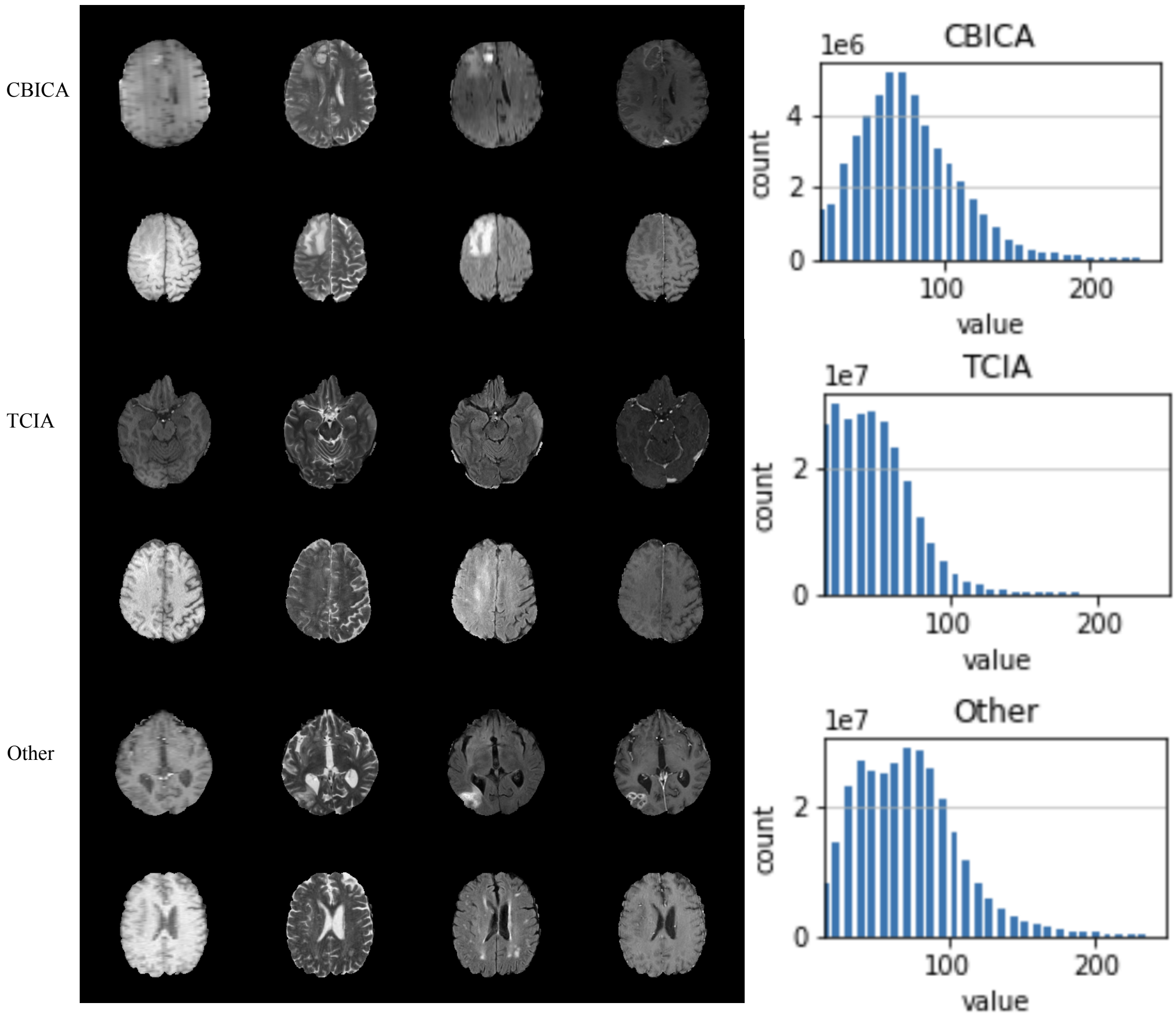

Supplementary Figure 7: Data heterogeneous of brain MRI data centers. Randomly selected real image samples (2 samples per center) and intensity histogram charts from three data centers used in the multi-modality data experiments.

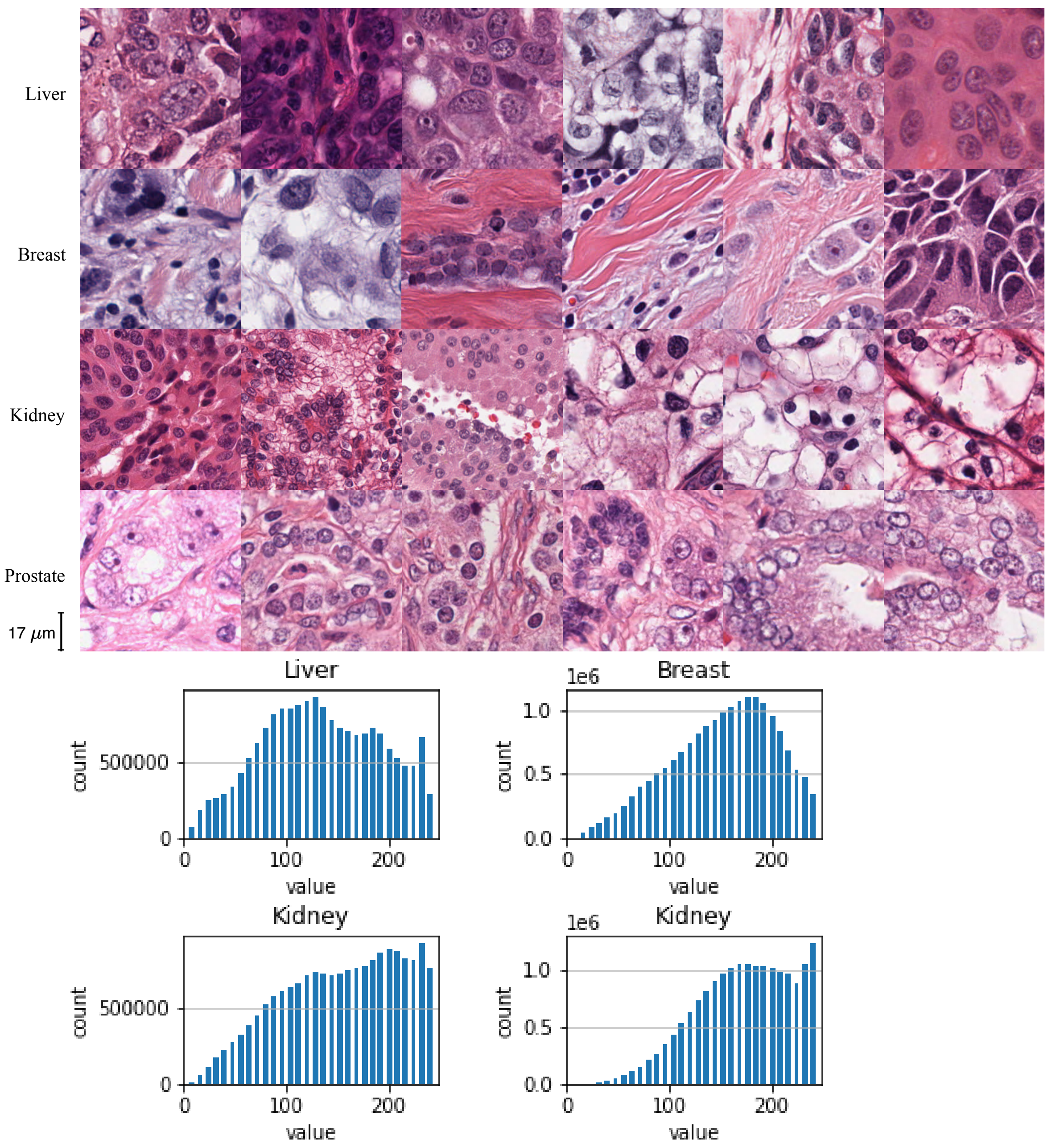

Supplementary Figure 8: Data heterogeneous of histopathology data centers. Randomly selected real image samples (6 samples per center) and intensity histogram charts from four different data centers (each center has one distinct organ) used in the continual-learning experiments.
