# Supplementary material for: Mining multi-center heterogeneous medical data with distributed synthetic learning
Source: Nat Commun. 2023 Sep 7;14:5510. doi: 10.1038/s41467-023-40687-y (PMC10484909; doi:10.1038/s41467-023-40687-y)
Supplement: Supplementary file 3 — Reporting Summary [file 41467_2023_40687_MOESM3_ESM.pdf]

nature portfolio

Corresponding author(s): Dimitris N. Metaxas

Last updated by author(s): Jun 30, 2023

Reporting Summary

Nature Portfolio wishes to improve the reproducibility of the work that we publish. This form provides structure for consistency and transparency in reporting. For further information on Nature Portfolio policies, see our Editorial Policies and the Editorial Policy Checklist.

Statistics

For all statistical analyses, confirm that the following items are present in the figure legend, table legend, main text, or Methods section.

n/a	Confirmed	
☐	☒	The exact sample size (*n*) for each experimental group/condition, given as a discrete number and unit of measurement
☐	☒	A statement on whether measurements were taken from distinct samples or whether the same sample was measured repeatedly
☐	☒	The statistical test(s) used AND whether they are one- or two-sided<br>*Only common tests should be described solely by name; describe more complex techniques in the Methods section.*
☒	☐	A description of all covariates tested
☒	☐	A description of any assumptions or corrections, such as tests of normality and adjustment for multiple comparisons
☐	☒	A full description of the statistical parameters including central tendency (e.g. means) or other basic estimates (e.g. regression coefficient) AND variation (e.g. standard deviation) or associated estimates of uncertainty (e.g. confidence intervals)
☐	☒	For null hypothesis testing, the test statistic (e.g. F, t, r) with confidence intervals, effect sizes, degrees of freedom and P value noted<br>*Give P values as exact values whenever suitable.*
☒	☐	For Bayesian analysis, information on the choice of priors and Markov chain Monte Carlo settings
☒	☐	For hierarchical and complex designs, identification of the appropriate level for tests and full reporting of outcomes
☒	☐	Estimates of effect sizes (e.g. Cohen's d, Pearson's r), indicating how they were calculated

Our web collection on statistics for biologists contains articles on many of the points above.

Software and code

Policy information about availability of computer code

Data collection	No software is used to collect data.
Data analysis	The source code of DSL and segmentation used in this study can be found at: https://github.com/tommy-qichang/DSL_All_Code

For manuscripts utilizing custom algorithms or software that are central to the research but not yet described in published literature, software must be made available to editors and reviewers. We strongly encourage code deposition in a community repository (e.g. GitHub). See the Nature Portfolio guidelines for submitting code & software for further information.

Data

Policy information about availability of data

All manuscripts must include a data availability statement. This statement should provide the following information, where applicable:

- Accession codes, unique identifiers, or web links for publicly available datasets
- A description of any restrictions on data availability
- For clinical datasets or third party data, please ensure that the statement adheres to our policy

The cardiac datasets include publicly avalable MM-WHS (https://zmiclab.github.io/zxh/0/mmwhs/, ASOCA (https://asoca.grand-challenge.org/), and CAT08(Schaap, M. et al. Standardized evaluation methodology and reference database for evaluating coronary artery centerline extraction algorithms. Medical Image Analysis 13, 701–714 (2009)).
The brain data are avaiable through BraTS 2018 Challenge https://www.med.upenn.edu/sbia/brats2018.html

The nuclei data are now part of the MoNuSeg training set (https://monuseg.grand-challenge.org/Data/)
The WHS masks for ASOCA and CAT08 data can be downloaded at https://github.com/tommy-qichang/DSL_All_Code/tree/main/data

Human research participants

Policy information about studies involving human research participants and Sex and Gender in Research.

Reporting on sex and gender	Such information has not been collected.
Population characteristics	Such information has not been collected.
Recruitment	Such information has not been collected.
Ethics oversight	Such information has not been collected.

Note that full information on the approval of the study protocol must also be provided in the manuscript.

Field-specific reporting

Please select the one below that is the best fit for your research. If you are not sure, read the appropriate sections before making your selection.

☒ Life sciences ☐ Behavioural & social sciences ☐ Ecological, evolutionary & environmental sciences

For a reference copy of the document with all sections, see nature.com/documents/nr-reporting-summary-flat.pdf

Life sciences study design

All studies must disclose on these points even when the disclosure is negative.

Sample size	Cardiac CTA experiments have 78 training sample subjects(11241 2D images) from WHS(20), ASOCA(32) and CAT08(26); 54 testing samples(8296 2D images)<br>BraTS18 related experiments have 168 training sample subjects(11349 2D images),42 testing sample subjects(2730 2D images)<br>Nuclei related experiments have 16 training sample subjects and 8 testing sample subjects.
Data exclusions	In preprocessing stage, we exclude all blank slices. In the downstream segmentation tasks, we exclude the image slices that don't have any mask (skull or any annotations).
Replication	We verified all experiments can be replicated using our source code.
Randomization	In the downstream segmentation task, we randomly select 20% samples from the training set to form a validation set for model selection.
Blinding	All our experiments are using public datasets which have been anonymized and de-identified. In such case, blinding may not be necessary as the data does not contain personally identifiable information that could introduce bias.

Reporting for specific materials, systems and methods

We require information from authors about some types of materials, experimental systems and methods used in many studies. Here, indicate whether each material, system or method listed is relevant to your study. If you are not sure if a list item applies to your research, read the appropriate section before selecting a response.

Materials & experimental systems

n/a	Involved in the study	
☒	☐	Antibodies
☒	☐	Eukaryotic cell lines
☒	☐	Palaeontology and archaeology
☒	☐	Animals and other organisms
☒	☐	Clinical data
☒	☐	Dual use research of concern

Methods

n/a	Involved in the study	
☒	☐	ChIP-seq
☒	☐	Flow cytometry
☒	☐	MRI-based neuroimaging
